# Injection network drivers of HIV prevention service utilization among people who inject drugs: results of a community‐based sociometric network cohort in New Delhi, India

**DOI:** 10.1002/jia2.26241

**Published:** 2024-04-17

**Authors:** Neia S. Prata Menezes, Shruti H. Mehta, Amy Wesolowski, Steven J. Clipman, Aylur K. Srikrishnan, Muniratnam S. Kumar, Katie J. C. Zook, Gregory M. Lucas, Carl Latkin, Sunil S. Solomon

**Affiliations:** ^1^ Department of Epidemiology Johns Hopkins University Bloomberg School of Public Health Baltimore Maryland USA; ^2^ Department of Medicine Johns Hopkins University School of Medicine Baltimore Maryland USA; ^3^ YR Gaitonde Centre for AIDS Research and Education Chennai India; ^4^ Department of Health, Behaviour and Society Johns Hopkins University Bloomberg School of Public Health Baltimore Maryland USA

**Keywords:** drug use, HIV prevention, HIV testing, harm reduction, LMIC, injection network

## Abstract

**Introduction:**

Injection drug networks may influence their network members’ health‐seeking behaviours. Using data from a sociometric injecting partner network of people who inject drugs (PWID) in New Delhi, India, we assessed the role of injecting partner (alter) behaviours on individual engagement in HIV prevention services.

**Methods:**

We enumerated injecting partner linkages among 2512 PWID using coupon referrals and biometric data from November 2017 to March 2020. Participants completed interviewer‐administered questionnaires and provided information on injection behaviours, injecting partners, HIV/hepatitis C (HCV) testing and service engagement. Multilevel multiple‐membership models (MMMM) evaluated individual PWID HIV testing, medication for opioid use disorder (MOUD) and syringe service engagement as a function of alter attributes, accounting for membership across multiple ego‐networks. Logistic regression models assessed parallel associations among socially proximal injecting peers, defined as PWID ≤3 path length from ego.

**Results:**

Median age was 26 years; 99% were male. PWID had median 2 injecting partners and 8 socially proximal peers; 14% reported HIV testing, 33% accessed MOUD and 13% used syringe services 6 months prior. In MMMM analyses, PWID with ≥1 versus 0 injecting partners who received HIV testing were significantly more likely to report HIV testing (adjusted odds ratio [aOR]: 2.27, 95% confidence interval [CI]: 1.68–3.16), MOUD (aOR: 1.99, 95% CI: 1.60–2.53) and syringe service use (aOR: 1.66, 95% CI: 1.21–2.39). We observed similar findings for individual MOUD and syringe service use. Having ≥1 versus 0 HIV‐positive partners was associated with decreased HIV testing and MOUD but increased syringe service use (aOR: 1.54, 95% CI: 1.09–2.17). PWID with ≥1 versus 0 socially proximal peers who used non‐sterile injection equipment reported increased HIV testing (aOR: 1.39, 95% CI: 1.01–1.92), MOUD (aOR: 1.40, 95% CI: 1.10–1.77) and syringe service use (aOR: 1.82, 95% CI: 1.23–2.68).

**Conclusions:**

We found differential associative relationships between individual HIV prevention service engagement and the health or risk behaviours of direct and indirect alters. Characterizing network exposure beyond direct injecting partnerships provided important context on possible mechanisms of behavioural influence. Findings could be leveraged to design peer‐based interventions that promote network diffusion of health‐seeking behaviours.

## 1 INTRODUCTION

Globally, injection drug use drives some of the fastest‐growing HIV epidemics [1]. The National AIDS Control Organization (NACO) in India estimates that 9.3% of people who inject drugs (PWID) are living with HIV [2]. Studies report substantial geographic heterogeneity in PWID HIV epidemics, with city‐level prevalence ranging from 6% to 45%, and incidence estimates ranging from 1.43 to 21.3 per 100 person‐years [3, 4, 5]. Data point to growing drug injection trends across cities like New Delhi, with heroin and/or diverted buprenorphine injection predominating [3, 6, 7]. Although NACO has increased coverage of PWID‐focused HIV‐prevention services, uptake remains suboptimal [8]. According to community‐derived samples of PWID in New Delhi (2016/17), 18% received HIV testing in the prior 6 months, and 52% and 35% received medication for opioid use disorder (MOUD) and accessed syringe services in the prior month, respectively [9].

Injection behaviours are often social, initiated and reinforced in an injection network, which comprises an interconnected group of PWID who inject together directly, or are indirectly connected to mutual injecting peers [10, 11]. Depending on the behaviours of injection network members, PWID may differentially engage in health‐seeking and risk‐taking behaviours. These influences may be heterogeneous for different behaviours. For example, injecting partner attributes (e.g. having multiple sexual partners, high‐risk injection practices) have been shown to predict concurrent PWID risk or health behaviours [12, 13]. Conversely, self‐reported condom use has been associated with positive condom‐use norms among network members [14]. In fact, network studies across various populations evince that people who report certain behaviours are more likely to report network members who engage in similar behaviours [15, 16, 17].

Mechanisms of behavioural influence vary and include modelling peer behaviours, social influence, differentially affiliating with those who practice similar behaviours, and/or establishing and maintaining network behavioural norms [18, 19, 20, 21]. Importantly, the degree of behavioural influence may vary by partnership type, strength or duration [22, 23, 24]. Evidence from India suggests that HIV, hepatitis C (HCV) and injection practices are negatively associated with having injecting peers who provide emotional support [25]. Conversely, data from high‐income settings indicate that unsafe injection practices are more common with injecting partners who are spouses or close friends [22, 24]. However, the relationship between injecting partners’ behaviours and individual health‐seeking practices, and the potential moderating effects of injecting partner relationships, are poorly understood.

While compelling evidence from other vulnerable populations, like men who have sex with men, suggests that peer influence may operate beyond direct ties [26, 27], studies among PWID from low‐ and middle‐income countries (LMICs) have been limited to egocentric network data. Egocentric data sample the individual (ego) and solicit information on their direct ties, relying solely on the ego's self‐reported network information [28, 29]. In contrast, sociometric data enumerate a set of individuals and all possible linkages between them, including direct and indirect ties [18]. Collecting these data is resource‐ and labour‐intensive making these data are rare [13, 29, 30]. Moreover, accounting for the interdependency between network ties in analyses poses methodological challenges [18, 31].

Using data from a sociometric cohort established through a community‐based sample of PWID in New Delhi, India, we evaluated whether individual PWID engagement in key HIV prevention services is associated with health‐seeking or high‐risk behaviours of direct injecting partners and/or indirect socially proximal peers.

## 2 METHODS

This study is nested within a dynamic, longitudinal sociometric cohort of PWID in New Delhi, India aimed to assess the impact of spatial‐, network‐ and individual‐level factors on HIV incidence among PWID [5, 32].

### 2.1 Participant recruitment

To construct the sociometric network, investigators recruited 10 “index” participants from New Delhi in 2017 who were ≥18 years old, provided written informed consent and reported a history of injecting drugs in the prior 24 months. Index participants were chosen to account for heterogeneity in drug injected, marital status, injection frequency and residential zip code. Index participants were asked to name and recruit injecting partners with whom they injected in the prior 30 days (based on similar eligibility criteria) using referral coupons [5, 32]. Network linkages (edges) were established between index and recruit, representing an injecting partnership. Similar to approaches taken with network studies, we assumed that all linkages were reciprocal [33]. Recruited alters were subsequently asked to recruit their own injecting partners using referral coupons. Partner referrals and linkages were tracked using network and coupon tracking software. Recruitment continued iteratively until the desired sample size was reached (*n* = 2500) and no additional network members were identified.

Participants were given a range of referral coupons (median = 1, range = 0–6) to reflect the number of injecting partners named, with no upper limit, and to ensure that recruitment reached network saturation. We used biometric data and network software to identify individuals recruited by multiple participants (i.e. duplicates). Duplicate participants were not enrolled again and did not provide additional information on injecting partners at baseline; however, their coupon data were used to establish cross‐network linkages [5, 32]. Participants were compensated for their study participation at the time of enrolment, with no additional compensation for duplicate recruitment, and provided incentives for each person they referred who completed study procedures. From November 2017 to March 2020, when study enrolment was halted due to the COVID‐19 pandemic, 2512 PWID were recruited, corresponding to 2640 ego‐alter dyads (5280 dyads, if accounting for participants’ separate roles as egos and alters). Overall, 75% of coupons were returned (2573/3455) (Table S1).

### 2.2 Data collection procedures

At baseline and follow‐up, participants completed electronic interviewer‐administered questionnaires and were asked to list venues where they injected in the prior 6 months. Blood samples for HIV and HCV testing were drawn at baseline and follow‐up visits. Rapid HIV testing was performed using three kits: Determine HIV‐1/2 (Alere Medical, USA), First Response HIV card test 1‐2‐O (Premier Medical, India) and Signal HIV‐1/2 (Arkray Healthcare, India). Rapid HCV antibody testing was performed using the Aspen HCV One Step Test Device (Aspen Diagnostics, India). This study used baseline data collected from November 2017 to March 2020.

### 2.3 Participant/ego‐level characteristics

All participants reported socio‐demographic information, including age, gender (analysed as male vs. female), marital status, education, employment history and homelessness. Participants also described HIV prevention service utilization in the prior 6 months (HIV testing, MOUD, syringe services) and lifetime HCV testing. Substance‐use information included age at first injection, drugs injected, frequency of injection, use of non‐sterile injection equipment in the prior 6 months and recent experiences with overdose. Participants also provided information on whether they injected at venue #40 in the prior 6 months, a commonly frequented venue previously linked to prevalent and incident HIV [5, 32]. Participants additionally reported experiences with incarceration and the number of male or female sexual partners in the prior 6 months. We measured hazardous alcohol use using the Alcohol Use Disorder Identification Test (AUDIT) scale [34] and depression using the Patient Health Questionnaire–9 [35].

### 2.4 Dyad‐level (ego‐alter) characteristics

“Index” and recruited participants (egos) provided socio‐demographic and behavioural information on injecting partners (alters), including relationship (friend, sibling/parent/other relative, spouse/sexual partner or other relationship [e.g. drug dealer, housemate]), number of years known, whether alter provides financial, material (food, shelter and/or drugs) or medical support, injection frequency with alter, use of non‐sterile injecting equipment with alter and alter's HIV/HCV status, if known. Participants also rated their trust in alters on a scale of 1 (low) to 10 (high). Trust scores (range 2–10) were dichotomized at the median value. Alter information was only provided by the ego who initially recruited them into the study.

### 2.5 Statistical analysis

#### 2.5.1 Multilevel multiple membership models

We applied multilevel multiple‐membership models (MMMM) to accommodate membership across multiple higher‐level units [36, 37, 38, 39]. We fit MMMM to evaluate individual HIV prevention service use as a function of the number of directly linked injecting partners engaged in health or risk behaviours, while accounting for membership across multiple ego‐networks (Figure S1). Injecting partners corresponded to alters directly linked to egos (i.e. one link/connection from ego). Explanatory variables were the number of directly linked injecting partners who reported using HIV prevention services (HIV testing, MOUD or syringe services), engaged in high‐risk substance use behaviours, or tested HIV or HCV positive at baseline, all parameterized as ≥1 versus 0. HIV testing, MOUD and syringe service outcomes were assessed separately. We assigned individual multiple membership weights that were inversely proportional to the total number of injecting partners in the sociometric network, summing to one [38]. Additionally, we allowed all dyadic relationships to be represented twice to reflect each participant's roles as ego and alter.

All models controlled for individual age, gender, marital status, education and log number of injecting partners *a priori*. Models also controlled for other individual factors if statistically significantly associated with the outcome (*p*<0.10). For HIV testing, we excluded 10 participants aware of their HIV‐positive serostatus and included participants who were diagnosed HIV positive at enrolment (yet reported HIV‐negative status). We did not adjust for cluster‐level variables given the high collinearity with individual‐level variables.

In secondary analyses, we restricted our sample to unique ego‐alter dyad pairs, where egos provided information on injecting partnership characteristics (e.g. relationship with alter, number of years known alter, frequency of injections with alter, trust in alter, alter's HIV/HCV status if known). Among these 2429 ego‐alter pairs (92% of dyads), we assessed whether partnership characteristics modified associations between injecting partner engagement in any HIV prevention service and individual engagement in HIV testing, MOUD or syringe services. We used cross‐classified multilevel logistic regression models, with random intercepts for ego and alter [31] since individuals belonged to only one higher‐level ego cluster, yet could serve as both egos and alters. In sensitivity analyses, we compared MMMM results to egocentric analytic approaches that aggregate personal network characteristics [31], excluded HIV‐positive participants at baseline and defined network exposure as the proportion of injecting partners.

#### 2.5.2 Evaluating ego‐network exposure from socially proximal peers

We enumerated socially proximal ego‐networks, such that all network peers who were ≤3 links from an ego were considered socially proximal alters. Our threshold for socially proximal ego‐networks was informed by U.S.‐based studies [15, 18]. As with MMMM analyses, all network composition variables were categorized as the ego having ≥1 versus 0 socially proximal alters (e.g. ≥1 vs. 0 alters who received HIV testing in the past 6 months). Logistic regression models assessed participant engagement in each HIV prevention service—HIV testing, MOUD and syringe services—as a function of each participant's aggregated socially proximal ego‐network characteristics (i.e. personal network exposure) [31]. Model adjustments were parallel to MMMM analyses, including *a priori* adjustment for log ego‐network size. To discern whether associations were primarily driven by direct (vs. indirect) linkages, we excluded direct injecting partnerships in sensitivity analyses. All MMMM were constructed using the brm command in R (*R Foundation for Statistical Computing*, 2020. Vienna, Austria), and model parameters were estimated using Markov chain Monte Carlo sampling [40, 41, 42]. All other models were fit using Stata 14 (StataCorp, 2015, College Station, TX).

#### 2.5.3 Ethical clearance

The study protocol was approved by Institutional Review Boards at Johns Hopkins Medicine (IRB00110421) and the YR Gaitonde Centre for AIDS Research and Education in India (YRG292). All participants provided written informed consent.

## 3 RESULTS

### 3.1 Participant/ego characteristics

**Figure 1:**
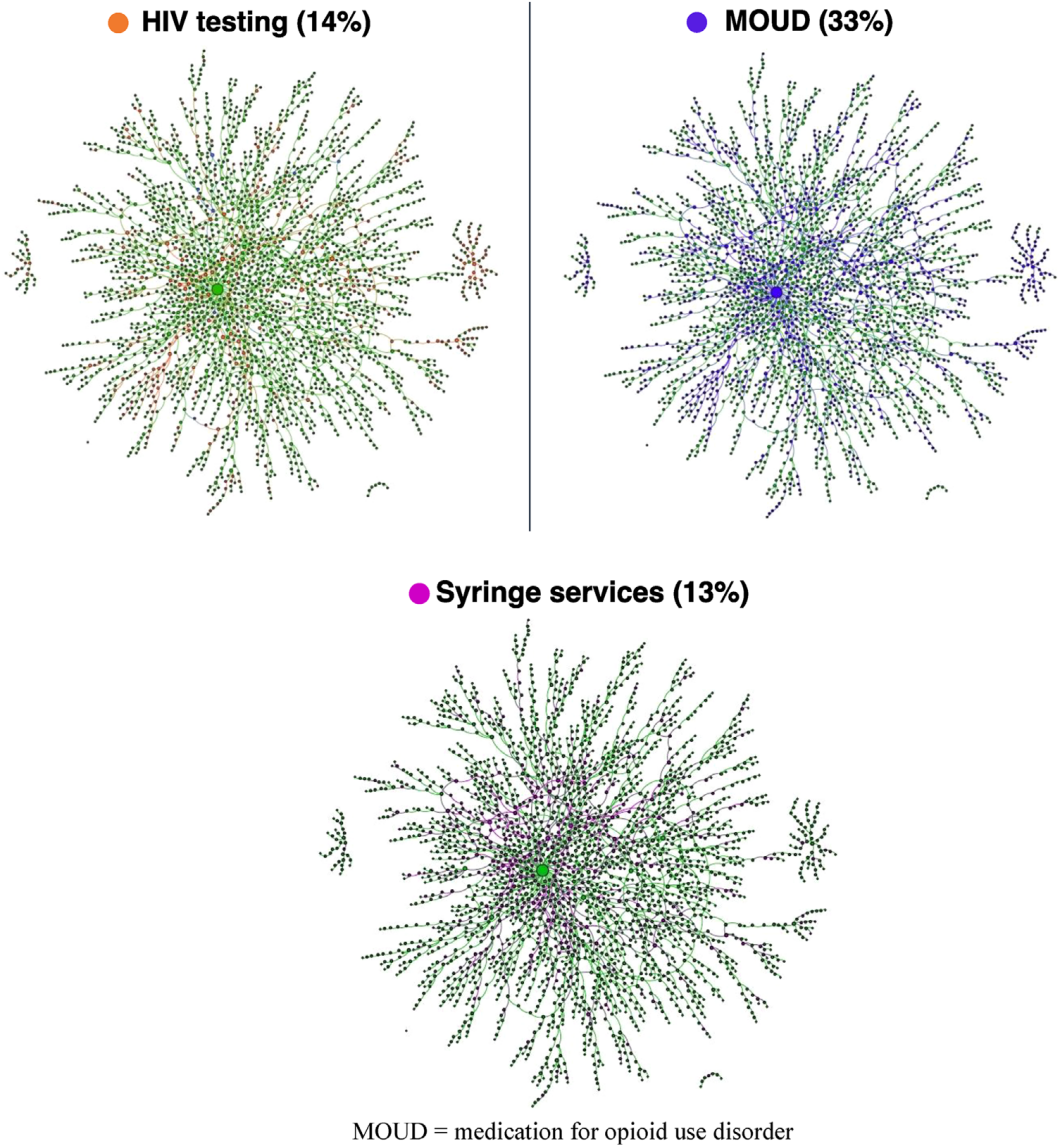
Injecting drug network of PWID in New Delhi, India, showcasing (a) HIV testing, (b) MOUD and (c) syringe service utilization in the prior 6 months.

Across 2512 participants, median age was 26 years (IQR 22–34), and 99.1% were male (Table 1). Approximately 37.3% (*n* = 938) tested HIV positive at baseline, including 10 participants who were aware of their HIV‐positive serostatus at baseline. Ninety‐nine percent reported injecting drugs in the prior 6 months, 67% reported injecting daily and 46.9% reported use of non‐sterile injection equipment in the prior 6 months. Buprenorphine was the primary drug injected (72.4%), followed by a combination of heroin and buprenorphine (22.2%). In the prior 6 months, 14.2% tested for HIV, 32.9% accessed MOUD and 13.5% received syringe services (Figure 1).

**Table 1:** Baseline demographic, network, substance use and HIV prevention characteristics in an injecting partner network of PWID in New Delhi, India

Baseline ego characteristics	*N* (%)
Total sample	2512
**Demographic characteristics**	
Age (median, IQR)	26 (22–34)
Gender	
Male	2489 (99.1)
Female	20 (0.8)
Hijra	3 (0.1)
Marital status	
Single/Widowed/Divorced	1800 (71.7)
Married/In partnership	708 (28.2)
*Missing*	4 (0.2)
HIV status	
HIV negative	1574 (62.7)
HIV positive	928 (36.9)
Known HIV positive	10 (0.4)
HCV status	
HIV negative	878 (35.0)
HIV positive	1633 (65.0)
Education	
No schooling	754 (30.0)
Primary school (Grades 1–5)	618 (24.6)
Secondary school (Grades 6–10) or above	1130 (45.0)
*Missing*	10 (0.4)
Employment in prior 12 months	
Unemployed	165 (6.6)
Earn daily wages	1545 (61.5)
Earn weekly or monthly wages	714 (28.4)
Other	84 (3.34)
*Missing*	4 (0.2)
Household monthly income in past year (median Rupees, IQR)	10,000 (7000–16,000)
Currently experiencing homelessness	
No	1754 (69.8)
Yes	754 (30.0)
*Missing*	4 (0.2)
**Injection drug network characteristics**	
No. of injecting partner ties in network (median, IQR)	2 (1–3)
**HIV prevention service utilization**	
Tested for HIV in prior 6 months (excluding known HIV positive)	
No	2147 (85.8)
Yes	355 (14.2)
*Reported known HIV‐positive status*	10
MOUD use in prior 6 months	
No	1685 (67.1)
Yes	827 (32.9)
Syringe services in prior 6 months	
No	2173 (86.5)
Yes	339 (13.5)
**Substance use and other risk factors**	
Ever tested for HCV	
No	2381 (93.8)
Yes	104 (4.1)
*Missing*	27 (1.1)
Age at first injection (median, IQR)	21 (18–26)
*Missing*	4 (0.02)
Types of drugs injected in prior 6 months	
Other	17 (0.7)
Heroin only	107 (4.3)
Buprenorphine only	1820 (72.4)
Heroin and buprenorphine	559 (22.2)
*Missing*	9 (0.4)
Frequency of drug injection in prior 6 months	
<Daily	817 (32.5)
Daily	1682 (67.0)
*Missing*	13 (0.52)
Used non‐sterile injection equipment in prior 6 months	
No	1319 (52.5)
Yes	1178 (46.9)
*Missing*	15 (0.6)
Passed needle after use in prior 6 months	1107 (44.1)
Used needle after someone else in prior 6 months	1114 (44.4)
Alcohol use in prior 6 months	
None	1525 (60.7)
Low/moderate	232 (9.2)
Harmful/hazardous	450 (17.9)
Dependence	304 (12.1)
*Missing*	1 (0.04)
Unintended drug overdose in past year	
Never or >1 year ago	1391 (55.4)
Yes	333 (13.3)
*Missing*	788 (31.4)
Injecting at venue #40 in prior 6 months	
No	1293 (51.5)
Yes	1219 (48.5)
Prison/Jail in past 6 months	
No	2436 (97.0)
Yes	76 (3.0)
No. of female sexual partners in past 6 months (median, IQR)	1 (1–1)
No. of male sexual partners in past 6 months (median, IQR)	1 (1–2.5)
Depression	
None/mild	1857 (73.9)
Moderate/severe	655 (26.1)

### 3.2 Ego‐network and ego‐alter dyad characteristics

Participants were directly linked to a median 2 injecting partners (IQR 1–3; range 1–61) and a median 8 socially proximal injecting peers (IQR 5–14; range 3–379). Overall, 26.7%, 54.6% and 28.7% of participants were directly linked to at least one injecting partner who received HIV testing, accessed MOUD or used syringe services in the prior 6 months, respectively (Table S2). Table 2 displays additional baseline relationship characteristics of direct alters and ego‐alter dyads. Across the 2429 (92%) ego‐alter dyads, most participants reported injecting partners who are friends (71.1%) and who they knew for ≥1 year (64.5%).

**Table 2:** Baseline injecting partnership relationship factors in an injecting partner network of PWID in New Delhi, India

Sample characteristics of alters in ego‐alter dyads	PWID participants, *N* (%)
***Ego‐alter relationship type*** ^a^	
Injecting partner alter is friend	
None	726 (28.9)
≥ 1 alter	1786 (71.1)
Injecting partner alter is family member (sibling, parent, relative)	
None	2493 (99.2)
≥ 1 alter	19 (0.8)
Injecting partner alter is spouse, sexual partner, boyfriend/girlfriend	
None	2509 (99.9)
≥ 1 alter	3 (0.1)
Other relationship type (dealer, work colleague, housemate)	
None	2445 (97.3)
≥ 1 alter	67 (2.7)
Has known alter for 1 or more years	
None	892 (35.5)
≥ 1 alter	1620 (64.5)
No. of years known alter (median, IQR)	4 (2–10)
Known alter for 5 or more years	
None	1558 (62.0)
≥ 1 alter	954 (38.0)
***Ego‐alter social support*** ^a^	
Trust scale score (median, IQR)	7 (5–8)
Trusts alter^b^	
None	1405 (55.9)
≥ 1 alter	1107 (44.1)
Alter would provide material support (drugs, food or shelter)	
None	1161 (46.2)
≥ 1 alter	1351 (53.8)
Alter would provide financial support	
None	996 (39.7)
≥ 1 alter	1516 (60.3)
Ego would discuss health‐related questions with alter	
None	2025 (80.6)
≥ 1 alter	487 (19.4)
***Ego‐alter injection behaviours* ^a^**	
No. of times injected with alters in prior 30 days (median, IQR)	15 (6–30)
Injected with alter more than 15 times in prior 30 days	
None	1525 (60.7)
≥ 1 alter	987 (39.3)
Used non‐sterile injection equipment with alter in past 30 days	
None	1778 (70.8)
≥ 1 alter	734 (29.2)

^a^ All ego‐alter dyad relationship characteristics are reported from perspective of ego (i.e. self‐reported by ego).

^b^ Trust scale—On a scale of 1–10, how much do you trust this injecting partner, with 1 meaning “I do not trust at all” and 10 meaning “I trust with my life?”. Continuous trust scale variable (range 2–10) was dichotomized so that those reporting values ≤6 indicated none/low trust levels, and those ≥7 indicated moderate to high trust levels.

### 3.3 HIV testing

**Figure 2:**
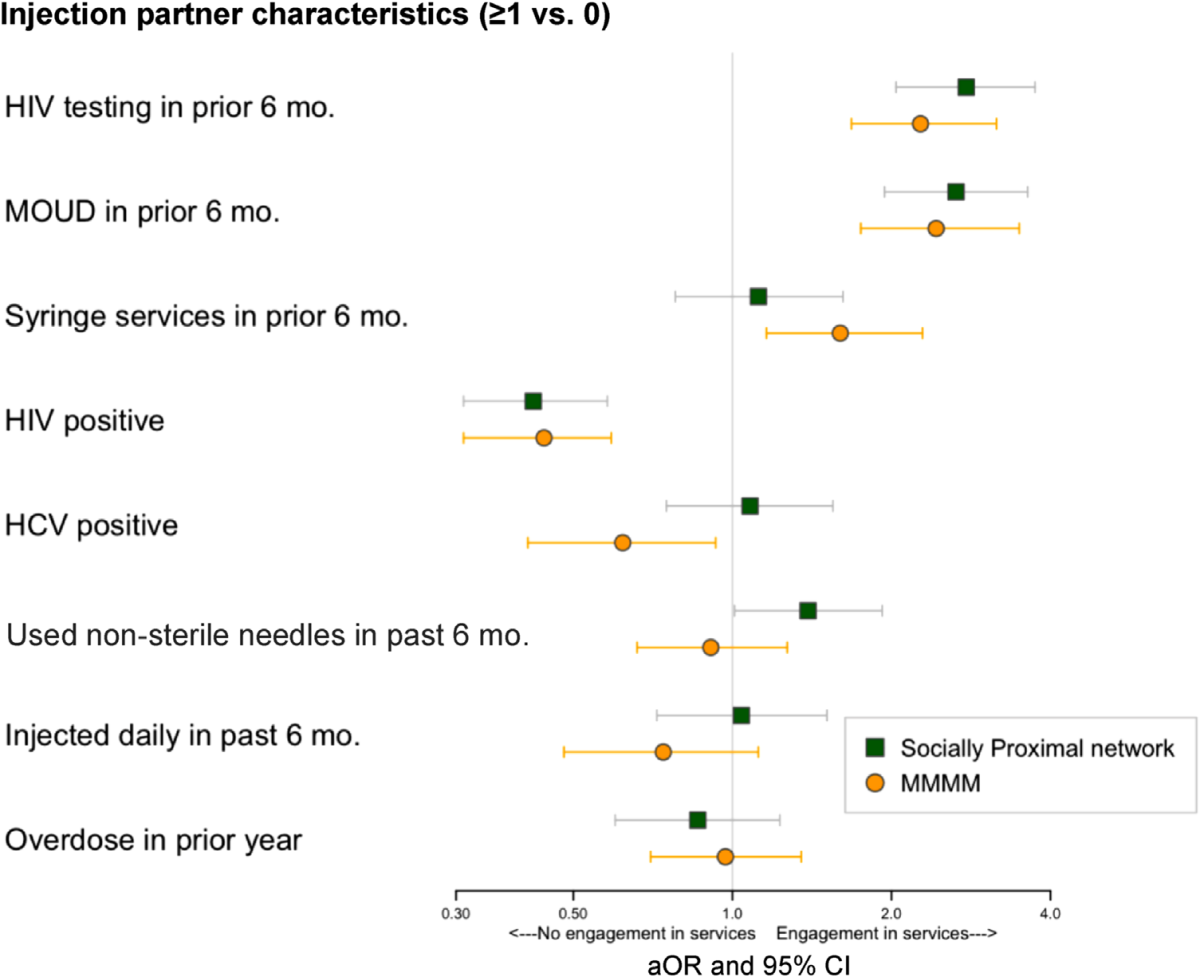
PWID engagement in *HIV testing* as a function of injection drug network members’ health behaviours, HIV/HCV status and substance use behaviours: Results of (a) Multilevel multiple membership models and (b) Egocentric analysis using socially proximal alters.

In MMMM analyses, HIV testing in the prior 6 months was associated with having ≥1 versus 0 injecting partners engaged in HIV testing (adjusted odds ratio [aOR]: 2.27, 95% confidence interval [CI]: 1.68–3.16), MOUD (aOR: 2.43, 95% CI: 1.75–3.49) and syringe services (aOR: 1.60, 95% CI: 1.16–2.29) (Figure 2 and Table S3). In contrast, we observed that having ≥1 versus 0 HIV‐positive injecting partners at baseline was associated with decreased odds of HIV testing in the prior 6 months (aOR: 0.44, 95% CI: 0.31–0.59). Similar associations were detected among PWID with HCV‐positive partners.

Results suggest that injection frequency modified the relationship between injecting partner and individual engagement in HIV prevention services. Specifically, the magnitude of association between having injecting partners who reported HIV prevention service utilization and HIV testing was greater among those who injected with partners ≤15 days (aOR: 3.75, 95% CI: 2.38–5.91) versus >15 days in the prior month (aOR: 1.61, 95% CI: 1.05–2.47) (*p*‐value for interaction effect <0.001) (Table 3).

**Table 3:** Associations between injecting partner use of HIV prevention services, and alter‐level engagement in HIV testing, MOUD and syringe services: assessing interaction effects by ego‐alter injecting partnership characteristics^a^

		HIV testing^b^	*p‐value for interaction*	MOUD^c^	*p‐value for interaction*	Syringe services^d^	*p‐value for interaction*
		*aOR (95% CI)*		*aOR (95% CI)*		*aOR (95% CI)*	
***Relationship type***							
Knows injecting partner for >1 year	Injecting partner HIV prevention service use in prior 6 months						
No	No	Ref.		Ref.		Ref.	
	Yes	4.27 (1.55–11.79)	—	1.80 (0.95–3.41)	—	0.72 (0.26–1.95)	—
Yes	No	Ref.		Ref.		Ref.	
	Yes	2.36 (1.70–3.29)	0.27	1.97 (1.58–2.45)	0.79	1.75 (1.29–2.39)	0.09
***Injection behaviours***							
Injected with injecting partner >15 times in prior 30 days	Injecting partner HIV prevention service use in prior 6 months						
No	No	Ref.		Ref.		Ref.	
	Yes	3.75 (2.38–5.91)	—	2.27 (1.69–3.05)	—	1.83 (1.20–2.80)	—
Yes	No	Ref.		Ref.		Ref.	
	Yes	1.61 (1.05–2.47)	<0.01	1.65 (1.24–2.19)	0.11	1.48 (1.00–2.20)	0.47

^a^ Injecting partnership characteristics (relationship type and injection behaviours) are reported based on ego's perspective. Multivariable, multilevel logistic regression models assess alter‐level engagement in HIV testing, MOUD or syringe services as a function of ego's use of any HIV prevention services in the prior 6 months. All service engagement is self‐reported by egos and alters.

^b^ HIV testing: Final models adjust for alter‐level age, gender, marital status and education *a priori*. Models also adjust for homelessness, ever tested for HCV, injection frequency, injecting at venue JB40 and prison within the last 6 months.

^c^ MOUD: Final models adjust for alter‐level age, gender, marital status, HIV status and education *a priori*. Models also adjust for ever tested for HCV, injection frequency, injecting at venue JB40 and alcohol use.

^d^ Syringe services: Final models adjust for alter‐level age, gender, marital status, HIV status and education *a priori*. Models also adjust for homelessness, ever tested for HCV, alcohol use, injecting at venue JB40 and depression.

Analyses of socially proximal ego‐networks yielded similar results—HIV testing was more common among those with socially proximal alters who reported HIV testing and MOUD use, but less common among those HIV positive. Unlike MMMM, we did not detect associations between having proximal alters engaged in syringe services and individual HIV testing, but we did detect associations with having ≥1 versus 0 proximal alters who used non‐sterile injection equipment in the prior 6 months (aOR: 1.39, 95% CI: 1.01–1.92).

### 3.4 MOUD

**Figure 3:**
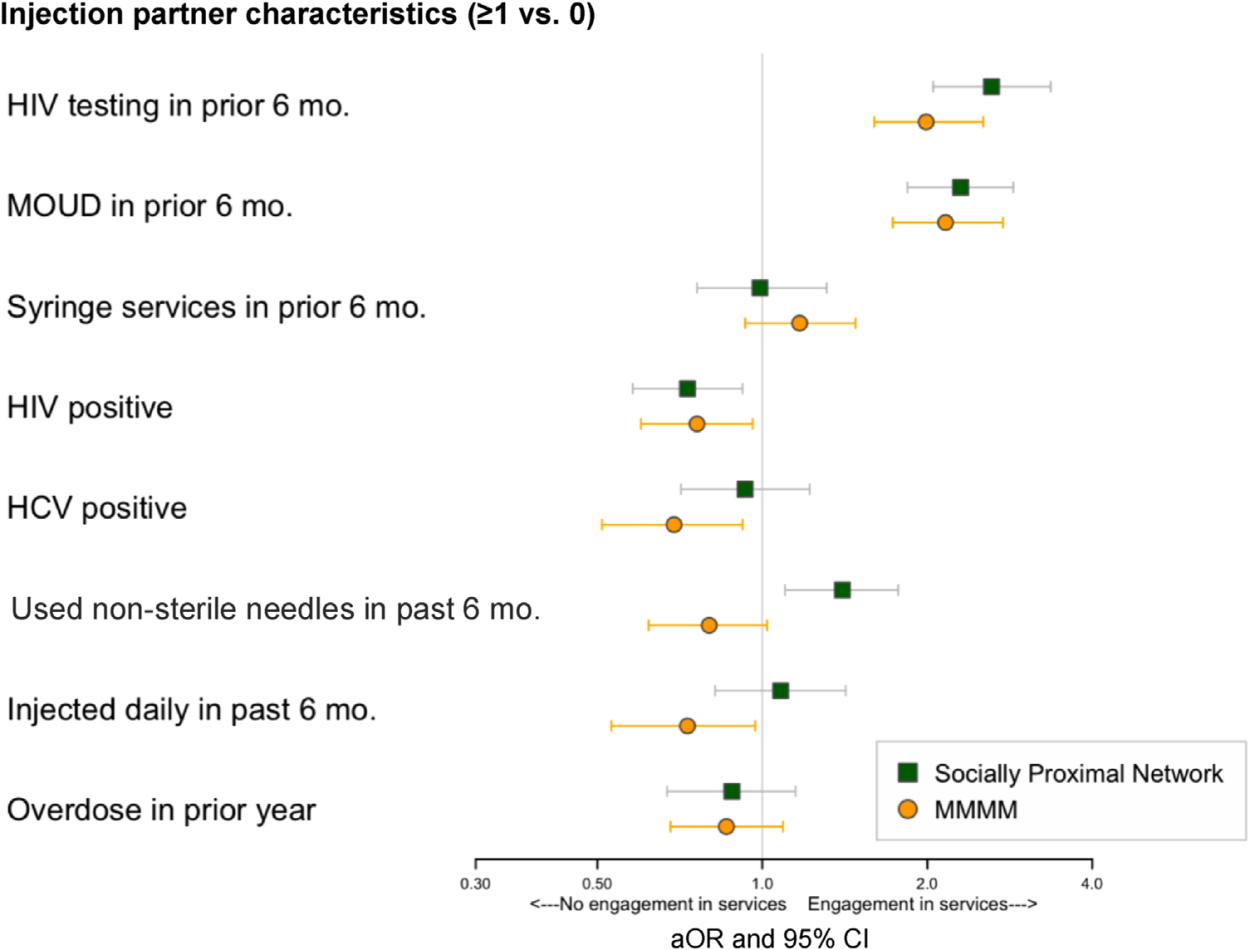
PWID engagement in *MOUD* as a function of injection drug network members’ health behaviours, HIV/HCV status and substance use behaviours: Results of (a) Multilevel multiple membership models and (b) Egocentric analysis using socially proximal alters.

Results evaluating MOUD use largely mirrored findings for HIV testing (Figure 3 and Table S4). Using MMMM, we observed higher MOUD engagement among PWID with injecting partners who reported HIV testing (aOR: 1.99, 95% CI: 1.60–2.53) and MOUD use (aOR: 2.16, 95% CI: 1.73–2.75) in the prior 6 months. Conversely, we observed lower engagement among PWID with HCV‐positive injecting partners (aOR: 0.69, 95% CI: 0.51–0.92) or with injecting partners who injected daily in the prior 6 months (aOR: 0.73, 95% CI: 0.53–0.97).

Using socially proximal ego‐networks, we observed that PWID with ≥1 versus 0 socially proximal alters engaged in HIV testing (aOR: 2.62, 95% CI: 2.05–3.36) or MOUD (aOR: 2.30, 95% CI: 1.84–2.87) were more likely to report MOUD use in the prior 6 months, and those with HIV‐positive injecting partners were less likely to engage in MOUD (aOR: 0.76, 95% CI: 0.60–0.96). However, having socially proximal alters who used non‐sterile injection equipment in the prior 6 months was linked to increased MOUD use (aOR: 1.40, 95% CI: 1.10–1.77).

### 3.5 Syringe services

**Figure 4:**
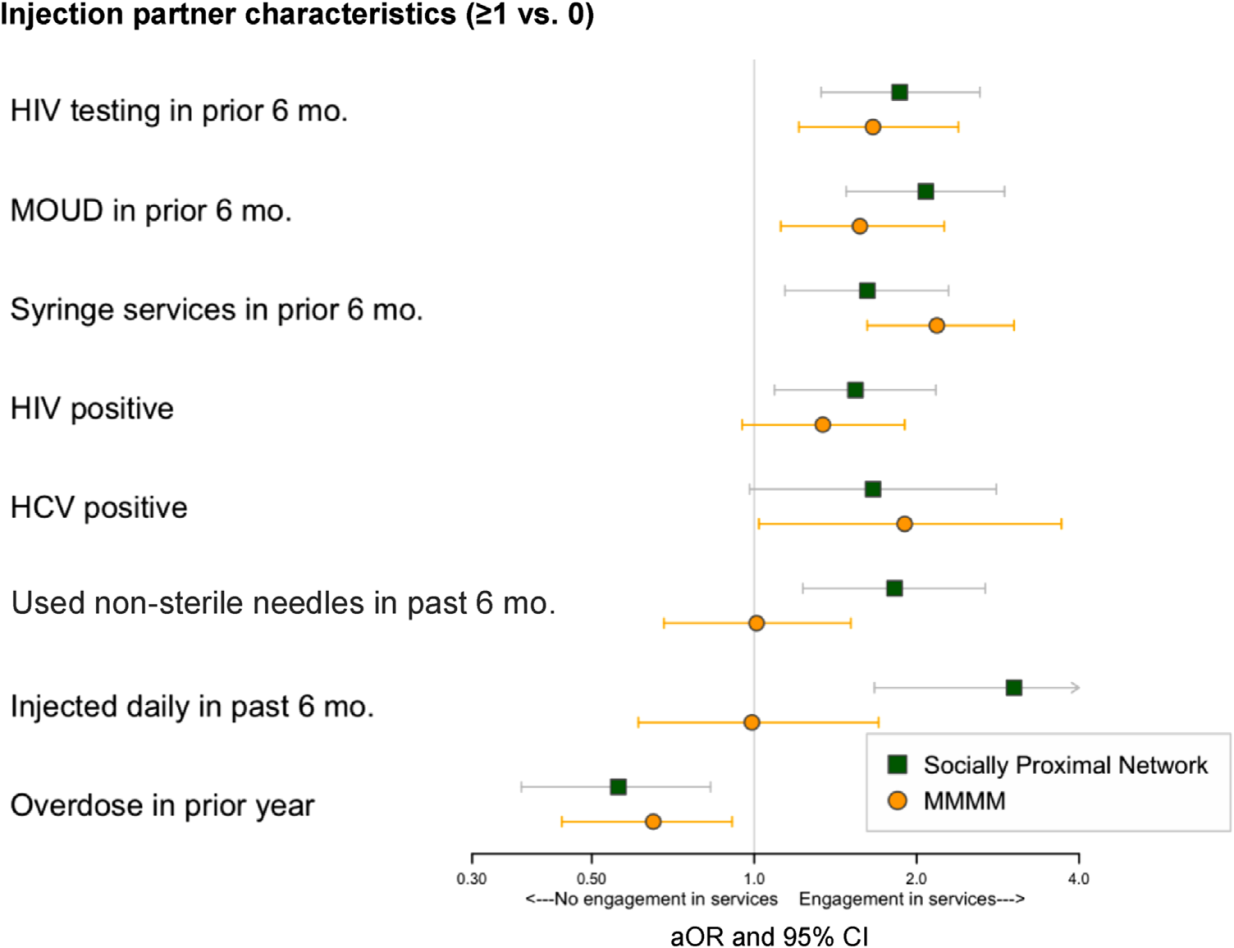
PWID engagement in *Syringe services* as a function of injection drug network's health behaviours, HIV/HCV status and substance use behaviours: Results of (a) Multilevel multiple membership models and (b) Egocentric analysis using socially proximal alters.

MMMM approaches suggest that having injecting partners engaged in HIV testing (aOR: 1.66, 95% CI: 1.21–2.39) or MOUD (aOR: 1.57, 95% CI: 1.12–2.25) was linked to PWID syringe service utilization (Figure 4 and Table S5). Having one HCV‐positive injecting partner was associated with PWID syringe service use (aOR: 1.90, 95% CI: 1.02–3.71). MMMM analyses also suggest decreased syringe service use among PWID with injecting partners who experienced recent drug overdose (aOR: 0.65, 95% CI: 0.44–0.91). There were no statistically significant associations between having injecting partners who were HIV positive, used non‐sterile injection equipment or injected daily.

Findings using socially proximal ego‐networks were predominantly analogous to MMMM results. However, analyses of socially proximal ego‐networks indicate increased syringe service utilization among PWID with socially proximally alters who were HIV positive (aOR: 1.62, 95% CI: 1.14–2.29), reported using non‐sterile injecting equipment (aOR: 1.82, 95% CI: 1.23–2.68) or injected daily (aOR: 3.03, 95% CI: 1.67–5.51) in the prior 6 months.

### 3.6 Sensitivity analyses

All sensitivity analyses of direct (Tables S6–S8) and socially proximal (Table S9) network connections yielded similar results. However, excluding direct injecting partnerships from socially proximal ego‐networks removed statistically significant associations between having proximal alters who used non‐sterile injecting equipment and individual HIV testing (aOR: 1.22, 95% CI: 0.88–1.69) or MOUD use (aOR: 1.26, 95% CI: 0.98–1.60) in the past 6 months.

## 4 DISCUSSION

In this large sociometric sample, we demonstrate strong positive associations between network member engagement in HIV preventive services and individual service utilization. While we cannot discern the mechanism of social influence, our study lends evidence for the diffusion of HIV prevention behaviours across PWID networks, reflected by associations of direct and indirect connections. Moreover, these associations appear to transcend specific services, highlighting that possible diffusion mechanisms may appertain to general access and utilization. Study findings could be leveraged to design and pilot future interventions aimed at promoting harm reduction and HIV prevention behaviours among PWID in the current Indian context.

Seminal network studies demonstrated that people who participate in certain behaviours are more likely to have close personal network associates who engage in similar behaviours [15, 43, 44, 45]. Prior studies have underscored similitude in risk and substance use behaviours among PWID injecting partners [46, 47]. Our data have been used to demonstrate the role of direct and indirect network ties in HIV and HCV transmission [32]. Our study adds to the existing literature by demonstrating that behavioural similarities between injecting partners extend to HIV prevention service engagement. Additionally, stronger associations were evident between HIV testing and MOUD use. Indeed, compared to individuals not engaged in either service, PWID who accessed HIV testing and MOUD exhibited fewer high‐risk behaviours and were more likely to use other services (e.g. HCV testing). These individuals were also less likely to inject daily, be HCV positive, report depression or be homeless, suggesting they may face fewer endogenous or exogenous barriers to service utilization.

Network theories have cited social capital, role modelling and abiding by perceived social norms as possible mechanisms through which social networks influence behaviours [18, 48, 49, 50]. PWID may model behaviours of their peers based on perceived social norms and/or to maintain social ties, particularly if injecting partners simultaneously provide social support, as previously demonstrated [25, 51]. Injection frequency was the only partnership characteristic that modified individual and partner HIV prevention behaviours in our sample. More frequent injection may reflect stronger drug dependence, which has been linked to poor HIV service engagement in PWID [52].

Interestingly, we observed that PWID with HIV/HCV‐positive injecting partners were less likely to report HIV testing or MOUD use, yet more likely to report syringe service use, even after controlling for individual‐level factors. Negative associations may signal severe drug addiction, high need for services and/or high prevalence of high‐risk injection practices associated with HIV serostatus. Only 1% of HIV‐positive participants reported serostatus awareness, underscoring the need to communicate regarding their HIV/HCV status, testing and treatment access. Negative associations may also signal low awareness of HIV prevention behaviours and/or a young population [53]. Young PWID are less likely to frequent services and demonstrate higher vulnerability. Indeed, 50% of our sample injected drugs for fewer than 5 years [53]. By contrast, positive associations between partner HIV/HCV status and syringe service utilization likely reflect engagement in high‐risk behaviours associated with HIV/HCV acquisition. Syringe services may also be more readily accessible in locations where PWID congregate. Unlike HIV testing and MOUD services, which typically are delivered in stand‐alone facilities, syringe services in India are primarily delivered through outreach models [8]. Outreach services can remove barriers to service access, like transportation costs or healthcare‐related stigma [54, 55, 56].

Our data are one of few to illustrate that MMMM analyses highly reflect findings of direct ego‐networks, a more common analytic approach (Table S7). MMMM provide an alternative approach for analysing sociometric data that accounts for the interdependencies inherent in social network data. Additionally, we demonstrate that correspondence between PWID and network member behaviours extends beyond direct injecting partnerships to socially proximal network peers. Moreover, analyses of socially proximal ego‐networks revealed associations not captured in MMMM. While direct ties often drive behavioural influences or disease transmission [18], our data intimate that indirect relationships also impact behaviours and provide insight into network norms. Personal network exposure is often associated with behavioural norms; however, the exposure threshold necessary to adopt new behaviours may vary by individual [18, 57]. Differences in signals detected using direct versus indirect ties may point to opportunities to leverage proximal ego‐networks to understand personal network threshold effects and drive behaviour change.

Our study has several limitations. First, these data were collected from 2017 to 2020, a period that we believe accurately reflects network relations among PWID in New Delhi before COVID‐19‐associated lockdowns altered the nature of PWID networks. Anecdotal data from this setting indicate mass migration and disruptions to usual locations where PWID injected [58, 59]. While data collection did resume in late 2021, any findings with more recent data would reflect these post‐COVID changes, which is beyond the scope of this analysis. Moreover, sociometric data such as these are rare from any setting much less an LMIC setting. Our research remains grounded in theoretical frameworks and models (behaviour modelling, social norms, network diffusion) [19, 21, 60] that remain applicable today.

Second, we assumed reciprocal network ties, a common assumption which may underestimate associations. Similarly, we assumed equivalent relationship ties, but some injecting partners may have greater influence over behaviours than others [61, 62]. Although we assessed differences in injection partnership characteristics, we may have been insufficiently powered. Third, 25% of coupons given to recruit injecting partners were not returned, signalling that our network may be missing individuals or cross‐network linkages. Indeed, our data suggest that individuals who successfully recruited participants tended to be older, HIV/HCV positive, engaged in HIV prevention services and less likely to inject daily. Nevertheless, this rate of missingness is lower than what is commonly observed in network studies. In our study, participants were incentivized for peer referrals to maximize the number of coupons returned during network recruitment. However, it is possible that incentives were insufficient which has been cited as a potential impediment to peer‐referral recruitment among PWID in India [63]. Other limitations include self‐reported service utilization and lack of information on distance to services. Finally, our data are cross‐sectional and preclude us from making causal claims regarding social influences [64].

## 5 CONCLUSIONS

To our knowledge, this is among the largest sociometric samples from a LMIC. Our findings demonstrate strong relationships between health‐seeking and high‐risk behaviours of injecting partners and individual engagement in HIV prevention services among PWID in New Delhi, highlighting the potential to leverage injecting partner networks to promote health‐seeking behaviours. Contextualizing network exposure beyond direct injecting pairs may provide auxiliary guidance to understand the diffusion of behaviours and interventions across PWID networks. We also add to the applications of statistical methods for sociometric data analysis. Future studies may incorporate qualitative data collection to better understand injecting partnership formation and the role of injecting partners in promoting or hindering health‐seeking behaviours.

## COMPETING INTERESTS

SHM reports non‐financial support from Abbott Laboratories outside the submitted work. SSS reports receiving grants, personal fees and non‐financial support from Gilead Sciences and grants and non‐financial support from Abbott Laboratories outside the submitted work. All other authors declare no competing interests.

## AUTHORS’ CONTRIBUTIONS

NSPM conceptualized the research question, conducted data analysis and drafted the manuscript. SHM, AW, CL, GML and SSS assisted with data interpretation and manuscript development. SJC, AKS, MSK and KJCZ oversaw data collection and/or data management. SSS designed the original study and oversaw study coordination. All authors reviewed the manuscript and approved the submitted version.

## FUNDING

This work was supported by the National Institutes of Health (grant numbers R01DA041736, DP2DA040244, DP2DA056130, R01DA041034, K24DA035684 and T32AI102623).

## DISCLAIMER

The funders of the study had no role in study design, data collection, data analysis, data interpretation, writing of the report or the decision to submit for publication.

## Supporting information


**Figure S1**. Comparing data structures of hierarchical multilevel models and non‐hierarchical multilevel multiple membership models
**Table S1**. Differences in baseline demographic, health, and substance use characteristics between participants whose referral coupons were returned vs. not returned
**Table S2**. Baseline demographic, health, and substance use characteristics of (a) direct injecting partnerships and (b) socially proximal ego‐networks in a sociometric injecting drug network of PWID in New Delhi, India
**Table S3**. PWID HIV testing engagement as a function of injecting drug network members' health behaviors, HIV/HCV status, and substance use behaviors: (a) Multilevel multiple membership models (MMMM) using direct injecting partners and (b) Egocentric analysis using socially proximal network peers
**Table S4**. PWID MOUD engagement as a function of injecting drug network members' health behaviors, HIV/HCV status, and substance use behaviors: (a) Multilevel multiple membership models (MMMM) using direct injecting partners and (b) Egocentric analysis using socially proximal network peers
**Table S5**. PWID syringe service engagement as a function of injecting drug network members' health behaviors, HIV/HCV status, and substance use behaviors: (a) Multilevel multiple membership models (MMMM) using direct injecting partners and (b) Egocentric analysis using socially proximal network peers
**Table S6**. HIV testing, MOUD, and syringe service engagement as a function of injecting drug network members' health behaviors, HIV/HCV status, and substance use behaviors: MMMM sensitivity analysis, using aggregated direct ego‐networks
**Table S7**. HIV testing, MOUD, and syringe service use as a function of health and risk behaviors, and HIV/HCV status, of direct injecting partners: MMMM sensitivity analysis, excluding known HIV‐positive participants at baseline (n = 10)
**Table S8**. HIV testing, MOUD, and syringe service use as a function of health and risk behaviors, and HIV/HCV status, of direct injecting partners: MMMM sensitivity analysis, defining network exposure as proportion of injecting partners
**Table S9**. HIV testing, MOUD, and syringe service engagement as a function of injecting drug network members' health behaviors, HIV/HCV status, and substance use behaviors: Sensitivity analysis, excluding direct injecting partnerships from socially proximal ego‐networks

## Data Availability

The data that support the findings of this study are available from SSS (sss@jhmi.edu) upon reasonable request.
