# Supplementary material for: Injection network drivers of HIV prevention service utilization among people who inject drugs: results of a community‐based sociometric network cohort in New Delhi, India
Source: J Int AIDS Soc. 2024 Apr 17;27(4):e26241. doi: 10.1002/jia2.26241 (PMC11024448; doi:10.1002/jia2.26241)
Supplement: Supplementary file 1 — Figure S1. Comparing data structures of hierarchical multilevel models and non‐hierarchical multilevel multiple membership models Table S1. Differences in baseline demographic, health, and substance use characteristics between participants whose referral coupons were returned vs. not returned Table S2. Baseline demographic, health, and substance use characteristics of (a) direct injecting partnerships and (b) socially proximal ego‐networks in a sociometric injecting drug network of PWID in New Delhi, India Table S3. PWID HIV testing engagement as a function of injecting drug network members' health behaviors, HIV/HCV status, and substance use behaviors: (a) Multilevel multiple membership models (MMMM) using direct injecting partners and (b) Egocentric analysis using socially proximal network peers Table S4. PWID MOUD engagement as a function of injecting drug network members' health behaviors, HIV/HCV status, and substance use behaviors: (a) Multilevel multiple membership models (MMMM) using direct injecting partners and (b) Egocentric analysis using socially proximal network peers Table S5. PWID syringe service engagement as a function of injecting drug network members' health behaviors, HIV/HCV status, and substance use behaviors: (a) Multilevel multiple membership models (MMMM) using direct injecting partners and (b) Egocentric analysis using socially proximal network peers Table S6. HIV testing, MOUD, and syringe service engagement as a function of injecting drug network members' health behaviors, HIV/HCV status, and substance use behaviors: MMMM sensitivity analysis, using aggregated direct ego‐networks Table S7. HIV testing, MOUD, and syringe service use as a function of health and risk behaviors, and HIV/HCV status, of direct injecting partners: MMMM sensitivity analysis, excluding known HIV‐positive participants at baseline (n = 10) Table S8. HIV testing, MOUD, and syringe service use as a function of health and risk behaviors, and HIV/HCV status, of direct [file JIA2-27-e26241-s001.docx]

# **APPENDIX: Supplemental Figures & Tables**

Table of Contents

[**APPENDIX: Supplemental Figures & Tables** 1](#_Toc161311863)

[**Figure 1.** Comparing data structures of hierarchical multilevel models and non-hierarchical multilevel multiple membership models 2](#_Toc161311864)

[**Table 1.** Differences in baseline demographic, health, and substance use characteristics between participants whose referral coupons were returned vs. not returned^Ψ^ 4](#_Toc161311865)

[**Table 2.** Baseline demographic, health, and substance use characteristics of (a) direct injecting partnerships and (b) socially proximal ego-networks in a sociometric injecting drug network of PWID in New Delhi, India 6](#_Toc161311866)

[**Table 3.** PWID HIV testing engagement as a function of injecting drug network members’ health behaviors, HIV/HCV status, and substance use behaviors: (a) Multilevel multiple membership models (MMMM) using direct injecting partners and (b) Egocentric analysis using socially proximal network peers ^±^ 8](#_Toc161311867)

[**Table 4.** PWID MOUD engagement as a function of injecting drug network members’ health behaviors, HIV/HCV status, and substance use behaviors: (a) Multilevel multiple membership models (MMMM) using direct injecting partners and (b) Egocentric analysis using socially proximal network peers ^±^ 9](#_Toc161311868)

[**Table 5.** PWID syringe service engagement as a function of injecting drug network members’ health behaviors, HIV/HCV status, and substance use behaviors: (a) Multilevel multiple membership models (MMMM) using direct injecting partners and (b) Egocentric analysis using socially proximal network peers ^±^ 10](#_Toc161311869)

[**Table 6.** HIV testing, MOUD, and syringe service engagement as a function of injecting drug network members’ health behaviors, HIV/HCV status, and substance use behaviors: MMMM sensitivity analysis, using aggregated direct ego-networks^±^ 11](#_Toc161311870)

[**Table 7.** HIV testing, MOUD, and syringe service use as a function of health and risk behaviors, and HIV/HCV status, of direct injecting partners: MMMM sensitivity analysis, excluding known HIV-positive participants at baseline (n=10) ^±^ 12](#_Toc161311871)

[**Table 8.** HIV testing, MOUD, and syringe service use as a function of health and risk behaviors, and HIV/HCV status, of direct injecting partners: MMMM sensitivity analysis, defining network exposure as proportion of injecting partners 13](#_Toc161311872)

[**Table 9.** HIV testing, MOUD, and syringe service engagement as a function of injecting drug network members’ health behaviors, HIV/HCV status, and substance use behaviors: Sensitivity analysis, excluding direct injecting partnerships from socially proximal ego-networks^±^ 14](#_Toc161311873)

## **Figure 1.** Comparing data structures of hierarchical multilevel models and non-hierarchical multilevel multiple membership models

(a) Traditional hierarchical multilevel structure

(b) Multilevel multiple membership data structure

*Dashed lines signify that alters are members of multiple ego-network groups/clusters*

*Methodological description of Figure S1*

We applied multilevel multiple-membership models (MMMM), an extension of multilevel models that accommodate membership across multiple higher-level units (Supplemental Figure 1) ^[1-3]^. Traditional multilevel models assume a strictly hierarchical data structure, whereby there is little to no overlap in higher order groups. Conversely, MMMM operate by assigning individual weights equal to the proportional affiliation in each higher order group. Although not novel, application of MMMM to social network data are few, despite models being particularly useful when lower level alters are nested within multiple higher-level ego networks ^[4]^. Ignoring the multiple membership structure of social network data violates the assumption of independent errors in multilevel analysis, potentially resulting in an underestimation of higher-level effects, incorrect standard errors estimations, and invalid inferences ^[5]^.

**References**

1. Browne WJ, Goldstein H, Rasbash J. **Multiple membership multiple classification (MMMC) models**. *Statistical Modelling* 2001; 1(2):103-124.

2. Rasbash J, Browne WJ. **Non-hierarchical multilevel models**. In: *Handbook of multilevel analysis*: Springer; 2008. pp. 301-334.

3. Hill PW, Goldstein H. **Multilevel modeling of educational data with cross-classification and missing identification for units**. *Journal of Educational and Behavioral statistics* 1998; 23(2):117-128.

4. Tranmer M, Steel D, Browne WJ. **Multiple‐membership multiple‐classification models for social network and group dependences**. *Journal of the Royal Statistical Society: Series A (Statistics in Society)* 2014; 177(2):439-455.

5. Perry BL, Pescosolido BA, Borgatti SP. **Egocentric network analysis: Foundations, methods, and models***.* Cambridge university press; 2018.

## **Table 1.** Differences in baseline demographic, health, and substance use characteristics between participants whose referral coupons were returned vs. not returned^Ψ^

| *Baseline ego characteristics* | PWID who had all, or some, referral coupons returned  N (%) | PWID who did not have any referral coupons returned  N (%) | P-value * |
| --- | --- | --- | --- |
| Total Sample | 2573 (69.4) | 766 (30.5) |  |
| **Demographic characteristics** |  |  |  |
| Age (median, IQR) | 27 (22-35) | 25 (21-31) | <0.01 |
| Gender |  |  |  |
| Male | 1728 (99.3) | 757 (99.1) | 0.66 |
| Female | 13 (0.7) | 7 (0.9) |  |
| Marital status |  |  |  |
| Single/Widowed/Divorced | 1251 (72.0) | 547 (71.4) | 0.77 |
| Married/In partnership | 487 (28.0) | 219 (28.6) |  |
| HIV status ^+^ |  |  |  |
| HIV-negative | 1065 (61.1) | 506 (66.1) | 0.02 |
| HIV-positive | 677 (38.9) | 260 (33.9) |  |
| HCV status |  |  |  |
| HCV-negative | 581 (33.3) | 295 (38.5) | 0.01 |
| HCV-positive | 1161 (66.7) | 471 (61.5) |  |
| Household monthly income in past year (median, IQR) | 10,000 (7,000-15,000) | 10,000 (700-20,000) | 0.08 |
| Currently experiencing homelessness |  |  |  |
| No | 1208 (69.5) | 543 (70.9) | 0.49 |
| Yes | 530 (30.5) | 223 (29.1) |  |
| **HIV prevention service utilization** |  |  |  |
| Tested for HIV in prior 6 months  (excluding known HIV-positive) |  |  |  |
| No | 1065 (61.3) | 506 (66.5) | 0.01 |
| Yes | 672 (38.7) | 255 (33.5) |  |
| MOUD use in prior 6 months |  |  |  |
| No | 1082 (62.1) | 600 (78.3) | <0.01 |
| Yes | 660 (37.9) | 166 (21.7) |  |
| Syringe services in prior 6 months |  |  |  |
| No | 1448 (83.1) | 722 (94.3) | <0.01 |
| Yes | 294 (16.9) | 44 (5.7) |  |
| **Substance use and other risk factors** |  |  |  |
| Ever tested for HCV |  |  |  |
| No | 1645 (95.0) | 732 (97.7) | <0.01 |
| Yes | 87 (5.0) | 17 (2.3) |  |
| Age at first injection (median, IQR) | 21 (18-26) | 20 (18-25) | 0.08 |
| Types of drugs injected in prior 6 months |  |  |  |
| Other | 13 (0.8) | 4 (0.5) | 0.02 |
| Heroin only | 72 (4.2) | 35 (4.6) |  |
| Buprenorphine only | 1234 (71.0) | 583 (76.5) |  |
| Heroin and buprenorphine | 418 (24.1) | 140 (18.4) |  |
| Frequency of drug injection in prior 6 months |  |  |  |
| <Daily | 606 (34.9) | 210 (27.6) | <0.01 |
| Daily | 1129 (65.1) | 550 (72.4) |  |
| Used non-sterile injection equipment in prior 6 months |  |  |  |
| No | 915 (52.7) | 401 (52.9) | 0.94 |
| Yes | 820 (47.3) | 357 (47.1) |  |
| Alcohol use in prior 6 months |  |  |  |
| None | 1058 (60.8) | 466 (60.8) | <0.01 |
| Low/ moderate | 139 (8.0) | 93 (12.1) |  |
| Harmful/ hazardous | 311 (17.9) | 137 (17.9) |  |
| Dependence | 233 (13.4) | 70 (9.1) |  |
| Unintended drug overdose in past year ^∞^ |  |  |  |
| Never or >1 year ago | 785 (76.5) | 603 (86.9) | <0.01 |
| Yes | 241 (23.5) | 91 (13.1) |  |
| Injected at venue #40 in prior 6 months |  |  |  |
| No | 938 (53.9) | 355 (46.3) | <0.01 |
| Yes | 804 (46.1) | 411 (53.7) |  |
| Prison/Jail in past 6 months |  |  |  |
| No | 1675 (96.2) | 757 (98.8) | <0.01 |
| Yes | 67 (3.8) | 9 (1.2) |  |
| No. female sexual partners in past 6 months  (median, IQR) | 1 (1-1) | 1 (1-1) | 0.68 |
| No. male sexual partners in past 6 months  (median, IQR) | 1 (1-3) | 1 (1-2) | 0.32 |
| Depression |  |  |  |
| None/mild | 1288 (73.9) | 566 (73.9) | 0.98 |
| Moderate/severe | 454 (26.1) | 200 (26.1) |  |
|  |  |  |  |

^Ψ^ Four individuals were excluded from this analysis given that they did not receive referral coupons to recruit injecting partners, likely attributable to the study halting because of the COVID-19 pandemic.

* P-value assessing differences in group characteristics. Chi-square and t-tests were used to assess group differences between categorical and continuous variables, respectively.

^+^ HIV-positive category includes participants who reported being aware of their HIV-positive status at baseline (n=10).

^∞^ Data only captured for participants who provided information on their drug overdose experience (n=1724).

## **Table 2.** Baseline demographic, health, and substance use characteristics of (a) direct injecting partnerships and (b) socially proximal ego-networks in a sociometric injecting drug network of PWID in New Delhi, India

| *Sample characteristics of ego-networks* | PWID participants, N (%) |
| --- | --- |
| ***(a) Characteristics of direct ego-networks (1 path length from ego)*** |  |
| **Engagement in HIV prevention services (self-reported by alter)** |  |
| HIV testing in past 6 months |  |
| None | 1842 (73.3) |
| ≥ 1 alter | 670 (26.7) |
| MOUD use in past 6 months |  |
| None | 1140 (45.4) |
| ≥ 1 alter | 1372 (54.6) |
| Syringe services in past 6 months |  |
| None | 1791 (71.3) |
| ≥ 1 alter | 721 (28.7) |
| **HIV/HCV status (as measured during survey)** |  |
| HIV positive |  |
| None | 1052 (41.9) |
| ≥ 1 alter | 1460 (58.1) |
| HCV positive |  |
| None | 414 (16.5) |
| ≥ 1 alter | 2098 (83.5) |
| **Alter injection behaviors (self-reported by alter)** |  |
| Used non-sterile needles in past 6 months |  |
| None | 754 (30.0) |
| ≥ 1 alter | 1758 (70.0) |
| Injected daily in past 6 months |  |
| None | 452 (18.0) |
| ≥ 1 alter | 2060 (82.0) |
| Unintended drug overdose in the prior year |  |
| None | 1800 (71.7) |
| ≥ 1 alter | 712 (28.3) |
| ***(b) Characteristics of indirect ego networks (≤3 path lengths from ego)*** |  |
| **Engagement in HIV prevention services (self-reported by alter)** |  |
| HIV testing in past 6 months |  |
| None | 683 (27.2) |
| ≥ 1 alter | 1829 (72.8) |
| MOUD use in past 6 months |  |
| None | 1085 (43.2) |
| ≥ 1 alter | 1427 (56.8) |
| Syringe services in past 6 months |  |
| None | 1792 (71.3) |
| ≥ 1 alter | 720 (28.7) |
| **HIV/HCV status (as measured during survey)** |  |
| HIV positive |  |
| None | 1082 (43.1) |
| ≥ 1 alter | 1430 (56.9) |
| HCV positive |  |
| None | 439 (17.5) |
| ≥ 1 alter | 2073 (82.5) |
| **Alter injection behaviors (self-reported by alter)** |  |
| Used non-sterile needles in past 6 months |  |
| None | 787 (31.3) |
| ≥ 1 alter | 1725 (68.7) |
| Injected daily in past 6 months |  |
| None | 381 (15.2) |
| ≥ 1 alter | 2131 (84.8) |
| Unintended drug overdose in the prior year |  |
| None | 1462 (84.8) |
| ≥ 1 alter | 262 (15.2) |
|  |  |

^a^ Direct ego-networks enumerated such that each participant represented an ego and all injecting partners directly linked to ego were considered part of direct ego-network.

^b^ Indirect ego-networks enumerated such that each participant represented an ego and all network peers who were ≤3 links from the ego were considered socially proximal alters.

## **Table 3.** PWID HIV testing engagement as a function of injecting drug network members’ health behaviors, HIV/HCV status, and substance use behaviors: (a) Multilevel multiple membership models (MMMM) using direct injecting partners and (b) Egocentric analysis using socially proximal network peers ^±^

| Network exposure defined as the number of network members (direct injecting partners or socially proximal peers) in each category | MMMM *^a^* | | Socially proximal ego-network *^b^* | |
| --- | --- | --- | --- | --- |
|  | *N = 2502* | | *N = 2502* | |
|  | OR (95% CI) | aOR (95% CI) | OR (95% CI) | aOR (95% CI) |
| ***Service engagement*** |  |  |  |  |
| HIV testing in past 6 months |  |  |  |  |
| None | Ref. | Ref. | Ref. | Ref. |
| ≥ 1 | 3.00 (2.32 - 3.93) | 2.27 (1.68 - 3.16) | 2.79 (2.21 - 3.52) | 2.77 (2.04 - 3.74) |
| MOUD use in past 6 months |  |  |  |  |
| None | Ref. | Ref. | Ref. | Ref. |
| ≥ 1 | 3.10 (2.39 - 4.05) | 2.43 (1.75 - 3.49) | 3.12 (2.39 - 4.07) | 2.65 (1.94 - 3.62) |
| Syringe services in past 6 months |  |  |  |  |
| None | Ref. | Ref. | Ref. | Ref. |
| ≥ 1 | 1.54 (1.21 - 1.95) | 1.60 (1.16 - 2.29) | 1.29 (1.02 - 1.65) | 1.12 (0.78 - 1.62) |
| ***HIV/HCV status*** |  |  |  |  |
| HIV positive |  |  |  |  |
| None | Ref. | Ref. | Ref. | Ref. |
| ≥ 1 | 0.62 (0.50 - 0.79) | 0.44 (0.31 - 0.59) | 0.73 (0.58 - 0.91) | 0.42 (0.31 - 0.58) |
| HCV positive |  |  |  |  |
| None | Ref. | Ref. | Ref. | Ref. |
| ≥ 1 | 0.83 (0.62 - 1.12) | 0.62 (0.41 - 0.93) | 1.21 (0.89 - 1.65) | 1.08 (0.75 - 1.55) |
| ***Injecting behaviors*** |  |  |  |  |
| Used non-sterile needle in past 6 months |  |  |  |  |
| None | Ref. | Ref. | Ref. | Ref. |
| ≥ 1 | 1.23 (0.95 - 1.60) | 0.91 (0.66 - 1.27) | 1.63 (1.25 - 2.11) | 1.39 (1.01 - 1.92) |
| Injected daily in past 6 months |  |  |  |  |
| None | Ref. | Ref. | Ref. | Ref. |
| ≥ 1 | 0.98 (0.72 - 1.35) | 0.74 (0.48 - 1.12) | 1.28 (0.94 - 1.76) | 1.04 (0.72 - 1.51) |
| Drug overdose in past year ^d^ |  |  |  |  |
| None | Ref. | Ref. | Ref. | Ref. |
| ≥ 1 | 1.12 (0.86 - 1.46) | 0.97 (0.70 - 1.35) | 1.19 (0.90 - 1.57) | 0.73 (0.49 - 1.09) |
|  |  |  |  |  |

OR = unadjusted odds ratio; aOR = adjusted odds ratio, CI = confidence interval, HCV = hepatitis C virus

^±^ Final models adjust for ego-level age, gender, marital status, education, and log of ego network size a priori. Models also adjust for homelessness, ever tested for HCV, injection frequency, injecting at venue JB40, and prison within the last 6 months.

^a^ Multilevel multiple membership models (MMMM) were used to evaluate alter engagement in HIV prevention services as a function of the number of direct injecting partners (egos) engaged in services or high-risk injection practices, and the number of direct injecting partners (egos) who are HIV or HCV positive.

^b^ Socially proximal ego-networks were enumerated such that each participant represented an ego and all network peers who were ≤3 links from the ego were considered socially proximal alters.

^c^ Only among those who answered questions regarding experiences with unintended overdose (n = 1724).

## **Table 4.** PWID MOUD engagement as a function of injecting drug network members’ health behaviors, HIV/HCV status, and substance use behaviors: (a) Multilevel multiple membership models (MMMM) using direct injecting partners and (b) Egocentric analysis using socially proximal network peers ^±^

| Network exposure defined as the number of network members (direct injecting partners or socially proximal peers) in each category | MMMM *^a^* | | Socially proximal ego-network *^b^* | |
| --- | --- | --- | --- | --- |
|  | *N = 2512* | | *N = 2512* | |
|  | OR (95% CI) | aOR (95% CI) | OR (95% CI) | aOR (95% CI) |
| ***Service engagement*** |  |  |  |  |
| HIV testing in past 6 months |  |  |  |  |
| None | Ref. | Ref. | Ref. | Ref. |
| ≥ 1 | 2.69 (2.20 - 3.35) | 1.99 (1.60 - 2.53) | 2.60 (2.17 - 3.12) | 2.62 (2.05 - 3.36) |
| MOUD use in past 6 months |  |  |  |  |
| None | Ref. | Ref. | Ref. | Ref. |
| ≥ 1 | 2.77 (2.29 - 3.46) | 2.16 (1.73 - 2.75) | 2.51 (2.10 - 3.00) | 2.30 (1.84 - 2.87) |
| Syringe services in past 6 months |  |  |  |  |
| None | Ref. | Ref. | Ref. | Ref. |
| ≥ 1 | 1.39 (1.14 - 1.70) | 1.17 (0.93 - 1.48) | 1.25 (1.05 - 1.50) | 0.99 (0.76 - 1.31) |
| ***HIV/HCV status*** |  |  |  |  |
| HIV positive |  |  |  |  |
| None | Ref. | Ref. | Ref. | Ref. |
| ≥ 1 | 0.94 (0.79 - 1.13) | 0.76 (0.60 - 0.96) | 0.98 (0.83 - 1.16) | 0.73 (0.58 - 0.92) |
| HCV positive |  |  |  |  |
| None | Ref. | Ref. | Ref. | Ref. |
| ≥ 1 | 0.96 (0.76 - 1.23) | 0.69 (0.51 - 0.92) | 1.10 (0.88 - 1.37) | 0.93 (0.71 - 1.22) |
| ***Injection behaviors*** |  |  |  |  |
| Used non-sterile needle in past 6 months |  |  |  |  |
| None | Ref. | Ref. | Ref. | Ref. |
| ≥ 1 | 1.15 (0.94 - 1.40) | 0.80 (0.62 - 1.02) | 1.57 (1.30 - 1.89) | 1.40 (1.10 - 1.77) |
| Injected daily in past 6 months |  |  |  |  |
| None | Ref. | Ref. | Ref. | Ref. |
| ≥ 1 | 1.06 (0.83 - 1.36) | 0.73 (0.53 - 0.97) | 1.32 (1.05 - 1.66) | 1.08 (0.82 - 1.42) |
| Drug overdose in past year ^d^ |  |  |  |  |
| None | Ref. | Ref. | Ref. | Ref. |
| ≥ 1 | 0.96 (0.77 - 1.18) | 0.86 (0.68 - 1.09) | 1.18 (0.96 - 1.46) | 0.84 (0.62 - 1.13) |
|  |  |  |  |  |

OR = unadjusted odds ratio; aOR = adjusted odds ratio, CI = confidence interval, HCV = hepatitis C virus

^±^ Final models adjust for alter-level age, gender, marital status, education, HIV status, and log of alter network size a priori. Models also adjust for ever tested for HCV, injection frequency, injecting at venue JB40, and alcohol use.

^a^ Multilevel multiple membership models (MMMM) were used to evaluate alter engagement in HIV prevention services as a function of the number of direct injecting partners (egos) engaged in services or high-risk injection practices, and the number of direct injecting partners (egos) who are HIV or HCV positive.

^b^ Socially proximal ego-networks were enumerated such that each participant represented an ego and all network peers who were ≤3 links from the ego were considered socially proximal alters.

^c^ Only among those who answered questions regarding experiences with unintended overdose (n = 1724).

## **Table 5.** PWID syringe service engagement as a function of injecting drug network members’ health behaviors, HIV/HCV status, and substance use behaviors: (a) Multilevel multiple membership models (MMMM) using direct injecting partners and (b) Egocentric analysis using socially proximal network peers ^±^

| Network exposure defined as the number of network members (direct injecting partners or socially proximal alters) in each category | MMMM *^a^* | | Socially proximal ego-network *^b^* | |
| --- | --- | --- | --- | --- |
|  | *N = 2512* | | *N = 2512* | |
|  | OR (95% CI) | aOR (95% CI) | OR (95% CI) | aOR (95% CI) |
| ***Service engagement*** |  |  |  |  |
| HIV testing in past 6 months |  |  |  |  |
| None | Ref. | Ref. | Ref. | Ref. |
| ≥ 1 | 2.10 (1.63 - 2.69) | 1.66 (1.21 - 2.39) | 3.32 (2.63 - 4.20) | 1.86 (1.33 - 2.62) |
| MOUD use in past 6 months |  |  |  |  |
| None | Ref. | Ref. | Ref. | Ref. |
| ≥ 1 | 2.16 (1.68 - 2.83) | 1.57 (1.12 - 2.25) | 3.35 (2.54 - 4.41) | 2.08 (1.48 - 2.91) |
| Syringe services in past 6 months |  |  |  |  |
| None | Ref. | Ref. | Ref. | Ref. |
| ≥ 1 | 3.74 (2.91 - 4.95) | 2.18 (1.62 - 3.03) | 3.87 (3.05 - 4.89) | 1.62 (1.14 - 2.29) |
| ***HIV/HCV status*** |  |  |  |  |
| HIV positive |  |  |  |  |
| None | Ref. | Ref. | Ref. | Ref. |
| ≥ 1 | 2.39 (1.80 - 3.06) | 1.34 (0.95 - 1.90) | 3.32 (2.52 - 4.37) | 1.54 (1.09 - 2.17) |
| HCV positive |  |  |  |  |
| None | Ref. | Ref. | Ref. | Ref. |
| ≥ 1 | 4.95 (2.97 - 8.76) | 1.90 (1.02 - 3.71) | 4.02 (2.50 - 6.47) | 1.66 (0.98 - 2.81) |
| ***Injection behaviors*** |  |  |  |  |
| Used non-sterile needle in past 6 months |  |  |  |  |
| None | Ref. | Ref. | Ref. | Ref. |
| ≥ 1 | 2.32 (1.70 - 3.19) | 1.01 (0.68 - 1.51) | 3.68 (2.63 - 5.14) | 1.82 (1.23 - 2.68) |
| Injected daily in past 6 months |  |  |  |  |
| None | Ref. | Ref. | Ref. | Ref. |
| ≥ 1 | 2.01 (1.36 - 3.13) | 0.99 (0.61 - 1.70) | 5.64 (3.27 - 9.74) | 3.03 (1.67 - 5.51) |
| Drug overdose in past year ^d^ |  |  |  |  |
| None | Ref. | Ref. | Ref. | Ref. |
| ≥ 1 | 0.73 (0.53 - 0.96) | 0.65 (0.44 - 0.91) | 1.83 (1.40 - 2.38) | 0.45 (0.29 - 0.69) |
|  |  |  |  |  |

OR = unadjusted odds ratio; aOR = adjusted odds ratio, CI = confidence interval, HCV = hepatitis C virus

^±^ Final models adjust for ego-level age, gender, marital status, education, HIV status, and log of ego network size a priori. Models also adjust for homelessness, ever tested for HCV, alcohol use, injecting at venue JB40, and depression.

^a^ Multilevel multiple membership models (MMMM) were used to evaluate alter engagement in HIV prevention services as a function of the number of injecting partners (egos) engaged in services or high-risk injection behaviors, and the number of injecting partners (egos) who are HIV or HCV positive.

^b^ Socially proximal ego-networks were defined such that each participant represented an ego, and all network peers who were ≤3 links from the ego were considered socially proximal alters.

^c^ Only among those who answered questions regarding experiences with unintended overdose (n = 1724).

## **Table 6.** HIV testing, MOUD, and syringe service engagement as a function of injecting drug network members’ health behaviors, HIV/HCV status, and substance use behaviors: MMMM sensitivity analysis, using aggregated direct ego-networks^±^

| Network exposure defined as the number of direct injecting partners (alters) in each category | N (%) | HIV testing *^a^* | | MOUD use *^b^* | | Syringe services *^c^* | |
| --- | --- | --- | --- | --- | --- | --- | --- |
|  |  | *N = 2502* | | *N = 2512* | | *N = 2512* | |
|  |  | OR (95% CI) | aOR (95% CI) | OR (95% CI) | aOR (95% CI) | OR (95% CI) | aOR (95% CI) |
| ***Service engagement*** |  |  |  |  |  |  |  |
| HIV testing in past 6 months |  |  |  |  |  |  |  |
| None | 1842 (73.3) | Ref. | Ref. | Ref. | Ref. | Ref. | Ref. |
| ≥ 1 | 670 (26.7) | 2.86 (2.27 - 3.61) | 2.06 (1.60 - 2.66) | 2.54 (2.12 - 3.05) | 1.91 (1.56 - 2.34) | 2.04 (1.60 - 2.58) | 1.58 (1.20 - 2.08) |
| MOUD use in past 6 months |  |  |  |  |  |  |  |
| None | 1140 (45.4) | Ref. | Ref. | Ref. | Ref. | Ref. | Ref. |
| ≥ 1 | 1372 (54.6) | 2.99 (2.31 - 3.86) | 2.23 (1.67 - 2.97) | 2.64 (2.21 - 3.15) | 2.07 (1.69 - 2.52) | 2.11 (1.65 - 2.70) | 1.46 (1.09 - 1.94) |
| Syringe services in past 6 months |  |  |  |  |  |  |  |
| None | 1791 (71.3) | Ref. | Ref. | Ref. | Ref. | Ref. | Ref. |
| ≥ 1 | 721 (28.7) | 1.51 (1.19 - 1.91) | 1.51 (1.15 - 1.98) | 1.35 (1.13 - 1.62) | 1.17 (0.94 - 1.44) | 3.59 (2.84 - 4.54) | 2.04 (1.56 - 2.67) |
| ***HIV/HCV status*** |  |  |  |  |  |  |  |
| HIV positive |  |  |  |  |  |  |  |
| None | 1052 (41.9) | Ref. | Ref. | Ref. | Ref. | Ref. | Ref. |
| ≥ 1 | 1460 (58.1) | 0.63 (0.51 - 0.79) | 0.49 (0.37 - 0.63) | 0.94 (0.79 - 1.11) | 0.78 (0.64 - 0.96) | 2.26 (1.75 - 2.93) | 1.28 (0.95 - 1.72) |
| HCV positive |  |  |  |  |  |  |  |
| None | 414 (16.5) | Ref. | Ref. | Ref. | Ref. | Ref. | Ref. |
| ≥ 1 | 2098 (83.5) | 0.82 (0.62 - 1.10) | 0.66 (0.47 - 0.93) | 0.94 (0.75 - 1.17) | 0.70 (0.54 - 0.92) | 4.51 (2.70 - 7.54) | 1.72 (0.99 - 2.99) |
| ***Injection behaviors*** |  |  |  |  |  |  |  |
| Used non-sterile needle in past 6 months |  |  |  |  |  |  |  |
| None | 754 (30.0) | Ref. | Ref. | Ref. | Ref. | Ref. | Ref. |
| ≥ 1 | 1758 (70.0) | 1.22 (0.94 - 1.57) | 0.91 (0.69 - 1.22) | 1.13 (0.94 - 1.35) | 0.83 (0.67 - 1.02) | 2.17 (1.62 - 2.91) | 1.00 (0.71 - 1.39) |
| Injected daily in past 6 months |  |  |  |  |  |  |  |
| None | 396 (15.8) | Ref. | Ref. | Ref. | Ref. | Ref. | Ref. |
| ≥ 1 | 2116 (84.2) | 0.96 (0.71 - 1.31) | 0.77 (0.54 - 1.08) | 1.05 (0.83 - 1.31) | 0.76 (0.58 - 0.99) | 1.85 (1.27 - 2.70) | 0.93 (0.61 - 1.42) |
| Drug overdose in past year ^d^ |  |  |  |  |  |  |  |
| None | 1391 (55.4) | Ref. | Ref. | Ref. | Ref. | Ref. | Ref. |
| ≥ 1 | 333 (13.3) | 1.11 (0.86 - 1.44) | 0.97 (0.73 - 1.28) | 0.96 (0.79 - 1.16) | 0.88 (0.71 - 1.08) | 0.73 (0.55 - 0.97) | 0.68 (0.50 - 0.93) |
|  |  |  |  |  |  |  |  |

OR = unadjusted odds ratio; aOR = adjusted odds ratio, CI = confidence interval, HCV = hepatitis C virus

^±^ Direct ego-networks enumerated such that each participant represented an ego and all injecting partners directly linked to ego were considered part of direct ego-network.

*^a^* HIV testing: Final models adjust for ego-level age, gender, marital status, education, and log of ego network size a priori. Models also adjust for homelessness, ever tested for HCV, injection frequency, injecting at venue JB40, and prison within the last 6 months.

*^b^* MOUD: Final models adjust for ego-level age, gender, marital status, education, HIV status, and log of ego network size a priori. Models also adjust for ever tested for HCV, injection frequency, injecting at venue JB40, and alcohol use.

*^c^* Syringe services: Final models adjust for ego-level age, gender, marital status, education, HIV status, and log of ego network size a priori. Models also adjust for homelessness, ever tested for HCV, alcohol use, injecting at venue JB40, and depression.

^d^ Only among those who answered questions regarding experiences with unintended overdose (n = 1724)

## **Table 7.** HIV testing, MOUD, and syringe service use as a function of health and risk behaviors, and HIV/HCV status, of direct injecting partners: MMMM sensitivity analysis, excluding known HIV-positive participants at baseline (n=10) ^±^

| Network exposure defined as the number of egos (injecting partners) in each category | HIV testing *^a^* | MOUD *^b^* | Syringe services *^c^* |
| --- | --- | --- | --- |
|  | *N = 2502* | *N = 2502* | *N = 2502* |
|  | aOR (95% CI) | aOR (95% CI) | aOR (95% CI) |
| ***Ego service engagement*** |  |  |  |
| HIV testing in past 6 months |  |  |  |
| None | Ref. | Ref. | Ref. |
| ≥ 1 | 2.25 (1.66 - 3.13) | 2.01 (1.62 - 2.51) | 1.68 (1.22 - 2.34) |
| MOUD use in past 6 months |  |  |  |
| None | Ref. | Ref. | Ref. |
| ≥ 1 | 2.43 (1.75 - 3.45) | 2.16 (1.75 - 2.72) | 1.58 (1.13 - 2.32) |
| Syringe services in past 6 months |  |  |  |
| None | Ref. | Ref. | Ref. |
| ≥ 1 | 1.62 (1.17 - 2.29) | 1.17 (0.93 - 1.78) | 2.20 (1.63 - 3.06) |
| ***Ego HIV/HCV status*** |  |  |  |
| HIV positive |  |  |  |
| None | Ref. | Ref. | Ref. |
| ≥ 1 | 0.44 (0.32 - 0.60) | 0.76 (0.61 - 0.95) | 1.35 (0.95 - 1.91) |
| HCV positive |  |  |  |
| None | Ref. | Ref. | Ref. |
| ≥ 1 | 0.63 (0.42 - 0.96) | 0.68 (0.49 - 0.94) | 1.90 (1.05 - 3.67) |
| ***Ego injection behaviors*** |  |  |  |
| Used non-sterile needles in past 6 months |  |  |  |
| None | Ref. | Ref. | Ref. |
| ≥ 1 | 0.91 (0.66 - 1.27) | 0.81 (0.63 - 1.02) | 1.00 (0.68 - 1.49) |
| Injected daily in past 6 months |  |  |  |
| None | Ref. | Ref. | Ref. |
| ≥ 1 | 0.74 (0.48 - 1.12) | 0.73 (0.53 - 0.98) | 1.00 (0.59 - 1.80) |
| Drug overdose in past year |  |  |  |
| None | Ref. | Ref. | Ref. |
| ≥ 1 | 0.97 (0.70 - 1.35) | 0.86 (0.68 - 1.09) | 0.64 (0.44 - 0.92) |
|  |  |  |  |

OR = unadjusted odds ratio, aOR = adjusted odds ratio, CI = confidence interval, HCV = hepatitis C virus

^±^ Multilevel multiple membership models (MMMM) were used to evaluate alter engagement in HIV prevention services as a function of the number of injecting partners (egos) engaged in services or high-risk injection behaviors, and the number of injecting partners (egos) who are HIV or HCV positive.

^a^ HIV testing: Final models adjust for alter-level age, gender, marital status, education, and log of alter network size a priori. Models also adjust for homelessness, ever tested for HCV, injection frequency, injecting at venue JB40, and prison within the last 6 months.

^b^ MOUD: Final models adjust for alter-level age, gender, marital status, education, HIV status, and log of alter network size a priori. Models also adjust for ever tested for HCV, injection frequency, injecting at venue JB40, and alcohol use.

^c^ Syringe services: Final models adjust for alter-level age, gender, marital status, education, HIV-status, and log of alter network size a priori. Models also adjust for homelessness, ever tested for HCV, alcohol use, injecting at venue JB40, and depression.

## **Table 8.** HIV testing, MOUD, and syringe service use as a function of health and risk behaviors, and HIV/HCV status, of direct injecting partners: MMMM sensitivity analysis, defining network exposure as proportion of injecting partners

| Network exposure defined as the number of alters (injecting partners) in each category | HIV testing *^a^* | MOUD *^b^* | Syringe services *^c^* |
| --- | --- | --- | --- |
|  | *N = 2502* | *N = 2512* | *N = 2512* |
|  | aOR (95% CI) | aOR (95% CI) | aOR (95% CI) |
| ***Alter service engagement*** |  |  |  |
| HIV testing in past 6 months | 1.16 (1.10 - 1.23) | 1.13 (1.09 - 1.17) | 1.08 (1.03 - 1.14) |
| MOUD use in past 6 months | 1.16 (1.12 - 1.22) | 1.12 (1.09 - 1.15) | 1.07 (1.03 - 1.12) |
| Syringe services in past 6 months | 1.09 (1.04 - 1.16) | 1.02 (0.98 - 1.06) | 1.15 (1.09 - 1.20) |
| ***Alter HIV/HCV status*** |  |  |  |
| HIV positive | 0.90 (0.85 - 0.94) | 0.96 (0.93 - 0.99) | 1.03 (0.99 - 1.08) |
| HCV positive | 0.94 (0.90 - 0.98) | 0.96 (0.93 - 0.99) | 1.07 (1.03 - 1.13) |
| ***Alter injection behaviors*** |  |  |  |
| Used non-sterile needles in past 6 months | 0.99 (0.94 - 1.03) | 0.99 (0.96 - 1.01) | 1.01 (0.97 - 1.06) |
| Injected daily in past 6 months | 0.97 (0.93 - 1.02) | 0.96 (0.93 - 0.99) | 1.01 (0.96 - 1.05) |
| Drug overdose in past year | 1.00 (0.93 - 1.06) | 0.98 (0.94 - 1.02) | 0.91 (0.84 - 0.98) |
|  |  |  |  |

OR = unadjusted odds ratio, aOR = adjusted odds ratio, CI = confidence interval, HCV = hepatitis C virus

^±^ Multilevel multiple membership models (MMMM) were used to evaluate alter engagement in HIV prevention services as a function of the number of injecting partners (egos) engaged in services or high-risk injection behaviors, and the number of injecting partners (egos) who are HIV or HCV positive.

^a^ HIV testing: Final models adjust for alter-level age, gender, marital status, education, and log of alter network size a priori. Models also adjust for homelessness, ever tested for HCV, injection frequency, injecting at venue JB40, and prison within the last 6 months.

^b^ MOUD: Final models adjust for alter-level age, gender, marital status, education, HIV status, and log of alter network size a priori. Models also adjust for ever tested for HCV, injection frequency, injecting at venue JB40, and alcohol use.

^c^ Syringe services: Final models adjust for alter-level age, gender, marital status, education, HIV-status, and log of alter network size a priori. Models also adjust for homelessness, ever tested for HCV, alcohol use, injecting at venue JB40, and depression

## **Table 9.** HIV testing, MOUD, and syringe service engagement as a function of injecting drug network members’ health behaviors, HIV/HCV status, and substance use behaviors: Sensitivity analysis, excluding direct injecting partnerships from socially proximal ego-networks^±^

| Network exposure defined as the number of alters in each category | HIV testing *^a^* | | MOUD use *^b^* | | Syringe services *^c^* | |
| --- | --- | --- | --- | --- | --- | --- |
|  | *N = 2502* | | *N = 2512* | | *N = 2512* | |
|  | aOR (95% CI) Including direct alters | aOR (95% CI) Excluding direct alters | aOR (95% CI) Including direct alters | aOR (95% CI) Excluding direct alters | aOR (95% CI) Including direct alters | aOR (95% CI) Excluding direct alters |
| ***Service engagement*** |  |  |  |  |  |  |
| HIV testing in past 6 mo. |  |  |  |  |  |  |
| None | Ref. | Ref. | Ref. | Ref. | Ref. | Ref. |
| ≥ 1 | 2.77 (2.04 - 3.74) | 2.37 (1.78 – 3.16) | 2.62 (2.05 - 3.36) | 2.00 (1.60 – 2.50) | 1.86 (1.33 - 2.62) | 1.85 (1.35 – 2.54) |
| MOUD use in past 6 mo. |  |  |  |  |  |  |
| None | Ref. | Ref. | Ref. | Ref. | Ref. | Ref. |
| ≥ 1 | 2.65 (1.94 - 3.62) | 2.22 (1.61 – 3.08) | 2.30 (1.84 - 2.87) | 1.96 (1.56 – 2.47) | 2.08 (1.48 - 2.91) | 1.87 (1.31 – 2.68) |
| Syringe services in past 6 mo. |  |  |  |  |  |  |
| None | Ref. | Ref. | Ref. | Ref. | Ref. | Ref. |
| ≥ 1 | 1.12 (0.78 - 1.62) | 1.05 (0.75 – 1.46) | 0.99 (0.76 - 1.31) | 1.18 (0.92 – 1.51) | 1.62 (1.14 - 2.29) | 2.02 (1.46 – 2.81) |
| ***HIV/HCV status*** |  |  |  |  |  |  |
| HIV positive |  |  |  |  |  |  |
| None | Ref. | Ref. | Ref. | Ref. | Ref. | Ref. |
| ≥ 1 | 0.42 (0.31 - 0.58) | 0.45 (0.33 – 0.61) | 0.73 (0.58 - 0.92) | 0.73 (0.59 – 0.92) | 1.54 (1.09 - 2.17) | 1.46 (1.02 – 2.08) |
| HCV positive |  |  |  |  |  |  |
| None | Ref. | Ref. | Ref. | Ref. | Ref. | Ref. |
| ≥ 1 | 1.08 (0.75 - 1.55) | 0.90 (0.62 – 1.31) | 0.93 (0.71 - 1.22) | 1.04 (0.78 – 1.38) | 1.66 (0.98 - 2.81) | 1.76 (1.00 – 3.09) |
| ***Injection behaviors*** |  |  |  |  |  |  |
| Used non-sterile needle in past 6 mo. |  |  |  |  |  |  |
| None | Ref. | Ref. | Ref. | Ref. | Ref. | Ref. |
| ≥ 1 | 1.39 (1.01 - 1.92) | 1.22 (0.88 – 1.69) | 1.40 (1.10 - 1.77) | 1.26 (0.98 – 1.60) | 1.82 (1.23 - 2.68) | 1.64 (1.09 – 2.48) |
| Injected daily in past 6 mo. |  |  |  |  |  |  |
| None | Ref. | Ref. | Ref. | Ref. | Ref. | Ref. |
| ≥ 1 | 1.04 (0.72 - 1.51) | 1.07 (0.72 – 1.61) | 1.08 (0.82 - 1.42) | 1.21 (0.90 – 1.63) | 3.03 (1.67 - 5.51) | 1.30 (0.75 – 2.23) |
| Drug overdose in past year ^d^ |  |  |  |  |  |  |
| None | Ref. | Ref. | Ref. | Ref. | Ref. | Ref. |
| ≥ 1 | 0.73 (0.49 - 1.09) | 0.94 (0.68 – 1.31) | 0.84 (0.62 - 1.13) | 1.01 (0.79 – 1.28) | 0.45 (0.29 - 0.69) | 0.76 (0.53 – 1.07) |
|  |  |  |  |  |  |  |

^±^ Socially proximal networks include all alters directly or indirectly connected to ego, by up to 3 linkages. For each HIV prevention service, results present associations between socially proximal network characteristics and individual service use (a) including direct alters and (b) excluding direct alters.

*^a^* HIV testing: Final models adjust for ego-level age, gender, marital status, education, and log of ego network size a priori. Models also adjust for homelessness, ever tested for HCV, injection frequency, injecting at venue JB40, and prison within the last 6 months.

*^b^* MOUD: Final models adjust for ego-level age, gender, marital status, education, HIV status, and log of ego network size a priori. Models also adjust for ever tested for HCV, injection frequency, injecting at venue JB40, and alcohol use.

*^c^* Syringe services: Final models adjust for ego-level age, gender, marital status, education, HIV status, and log of ego network size a priori. Models also adjust for homelessness, ever tested for HCV, alcohol use, injecting at venue JB40, and depression.

^d^ Only among those who answered questions regarding experiences with unintended overdose (n = 1724). OR = unadjusted odds ratio; aOR = adjusted odds ratio, CI = confidence interval, HCV = hepatitis C virus
